# Enhanced intracellular delivery and antibacterial efficacy of enrofloxacin-loaded docosanoic acid solid lipid nanoparticles against intracellular *Salmonella*

**DOI:** 10.1038/srep41104

**Published:** 2017-01-23

**Authors:** Shuyu Xie, Fei Yang, Yanfei Tao, Dongmei Chen, Wei Qu, Lingli Huang, Zhenli Liu, Yuanhu Pan, Zonghui Yuan

**Affiliations:** 1 National Reference Laboratory of Veterinary Drug Residues (HZAU), Huazhong Agricultural University, Wuhan, Hubei 430070, China; 2 MOA Laboratory for Risk Assessment of Quality and Safety of Livestock and Poultry Products, Huazhong Agricultural University, Wuhan, Hubei 430070, China; 3 MAO Key Laboratory for Detection of Veterinary Drug Residues, Huazhong Agricultural University, Wuhan, Hubei 430070, China

## Abstract

Enrofloxacin-loaded docosanoic acid solid lipid nanoparticles (SLNs) with different physicochemical properties were developed to enhance activity against intracellular *Salmonella*. Their cellular uptake, intracellular elimination and antibacterial activity were studied in RAW 264.7 cells. During the experimental period, SLN-encapsulated enrofloxacin accumulated in the cells approximately 27.06–37.71 times more efficiently than free drugs at the same extracellular concentration. After incubation for 0.5 h, the intracellular enrofloxacin was enhanced from 0.336 to 1.147 μg/mg of protein as the sizes of nanoparticles were increased from 150 to 605 nm, and from 0.960 to 1.147 μg/mg of protein when the charge was improved from −8.1 to −24.9 mv. The cellular uptake was more significantly influenced by the size than it was by the charge, and was not affected by whether the charge was positive or negative. The elimination of optimal SLN-encapsulated enrofloxacin from the cells was significantly slower than that of free enrofloxacin after removing extracellular drug. The inhibition effect against intracellular *Salmonella CVCC541* of 0.24 and 0.06 μg/mL encapsulated enrofloxacin was stronger than 0.6 μg/mL free drug after all of the incubation periods and at 48 h, respectively. Docosanoic acid SLNs are thus considered as a promising carrier for intracellular bacterial treatment.

Salmonellae are Gram-negative bacilli that cause enteric diseases in a wide range of animals. They are typically acquired by the ingestion of contaminated food or water. Moreover, exposure to bacteria-infected animals can also pose a high risk of salmonellosis in humans^1^. It was reported that approximately 1.2 million people are infected with *Salmonella* spp. annually in the United States^2^,^3^. *Salmonella* spp. are facultative intracellular bacteria and have evolved many mechanisms to evade the phagocytic killing mechanism of the mammalian host and establish specialized intracellular niches, sequestered from the host immune system, to produce a chronic carrier state^4^. Therefore, salmonellosis is difficult to treat because most of the available antimicrobial drugs (e.g., penicillins, cephalosporins and aminoglycosides) exhibit poor cellular diffusion and intracellular retention^5^.

Enrofloxacin, a second generation of fluoroquinolones, is used as a veterinary medicine for the treatment of salmonellosis because of its strong antibacterial properties and effective diffusion across cells^6^. However, it has low retention performance in host cells when the extracellular concentration decreases. It was observed that cells with accumulated enrofloxacin released about 80–90% of the drug within 10 min after being placed in enrofloxacin-free medium^6^. This problem results in treatment failure, drug resistance, high incidence of relapse, and drug-induced organ toxicity with repeated, high doses of treatment. Therefore, the intracellular clearance of *Salmonella*, mainly in macrophages, requires novel therapeutic strategies.

Nanocarriers have the ability to accumulate in macrophages and other cells, which makes them potentially useful for the treatment of intracellular infections, including *Salmonella*^5^. In this respect, liposomes and polymeric nanoparticles have been reported to be effective carriers, increasing the intracellular accumulation of fluoroquinolones at the site of infection with reduced toxicity and side effects. Liposomal enrofloxacin was reportedly delivered to Anatolian shepherd dog monocytes, resulting in the more effective treatment of intracellular infections than when free drugs were used^7^,^8^. Multilamellar liposome encapsulated enrofloxacin, which contained cholesterol and dipalmitoyl phosphatidylcholine in a molar ratio of 1:1.2, also produced a higher concentration in Kangal dog blood cells and was a more effective treatment for intracellular *Staphylococcus aureus* infections than solution^9^. In another study, liposomes composed of 15 mg egg phosphatidylcholine and 35 mg cholesterol resulted in increased activity of enrofloxacin against *S. aureus* in Turkish shepherd dog neutrophils^10^.

The properties of nanoparticles could greatly affect the intracellular delivery and efficacy of treatments. Ciprofloxacin nano-niosomes of 300–600 nm were more phagocytosed by macrophages than vesicles of 160–300 nm and 600–1000 nm^11^, while another study revealed that the delivery efficacy of ciprofloxacin-loaded liposomes to rat alveolar macrophages after pulmonary administration was enhanced by an increase in particle size from 100 to 1000 nm and became constant over 1000 nm^12^,^13^. The surface charge is also thought to be one of the most important factors in determining the intracellular behavior of nanoparticles and their encapsulated drugs^14^. Therefore, the investigation of common and decisive characteristics of nanoparticles in mediating cellular uptake is very important in the development of nanoparticle carriers.

Solid lipid nanoparticles (SLNs), an alternative drug carrier system to liposomes and polymeric nanoparticles, have attracted increasing attention due to their biocompatibility, biodegradability, stability, low cost and ease of large scale production^15^, and thus might be a promising carrier for the treatment of intracellular infections. Our previous study showed that fatty acid SLNs were effective nanoparticle systems for the controlled release and enhanced bioavailability of enrofloxacin in mice^15^. In this study, a series of enrofloxacin-loaded docosanoic acid SLNs of different sizes and zeta potentials were prepared by using a hot homogenization and ultrasonication method. The influence factors of intracellular delivery efficacy of enrofloxacin-loaded docosanoic acid SLNs were studied to obtain the optimum SLNs, and the antibacterial activity of the satisfactory nanoparticles was evaluated using the intracellular *Salmonella* infection model.

## Results

### Physicochemical characteristics of different docosanoic acid SLNs

**Figure 1:**
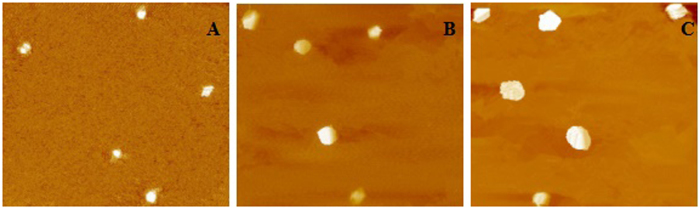
Photographs of atomic force microscopy (AFM) of different size of docosanoic acid SLNs (5 μm × 5 μm). (**A**) SLNs were prepared with 0.2 g enrofloxacin, 1.8 g docosanoic acid, and 30 ml 4% PVA by using 30 mm sonication probes with 80% amplitude; (**B**) SLNs were prepared with 0.2 g enrofloxacin, 1.8 g docosanoic acid, and 20 ml 2% PVA by using 6 mm sonication probes with 60% amplitude; (**C**) SLNs were prepared with 0.2 g enrofloxacin, 1.8 g docosanoic acid and 10 ml 1% PVA by using 3 mm sonication probes with 60% amplitude.

The preparation process and properties of the surfactant had a significant impact on the physicochemical characteristics of docosanoic acid SLNs. The mean size of docosanoic acid nanoparticles decreased from 605.0 to 150.1 nm as Polyvinyl alcohol (PVA) concentrations, aqueous phase volumes and the ultrasound probe diameter were increased from 1 to 4%, 10 to 30 mL and 3 to 30 mm, respectively (Table 1 and Fig. 1). The polydispersity index (PDI) ranged from 0.184 to 0.265 when PVA concentration was increased from 1% to 4%. The different charge of docosanoic acid nanoparticles was achieved by using varied concentrations of dimethyldioctadecyl ammonium chloride (DDAC) solution (Table 2). The zeta potential changed from −22.1 to 18.8 mv as the DDAC concentration was increased from 0 to 4%.

### Cellular uptake of encapsulated enrofloxacin

**Figure 2:**
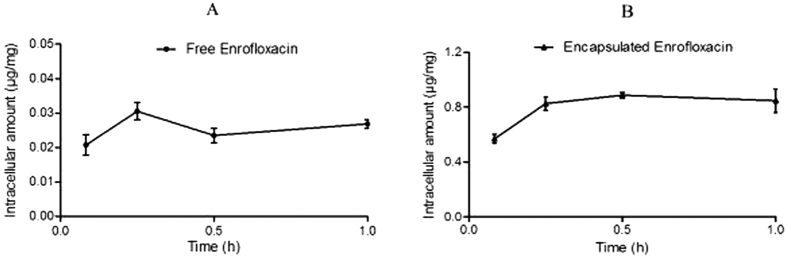
The cellular uptake kinetics of enrofloxacinin RAW.264.7 cells. (**A**) Free enrofloxacin; (**B**) Docosanoic acid encapsulated enrofloxacin. The enrofloxacin-loaded SLNs of 415 nm and −22.1 mv were prepared with 0.2 g enrofloxacin, 1.8 g fatty acid and 20 ml 2% PVA.

The accumulation of encapsulated and free enrofloxacin in cells exhibited a clear and significant difference (Fig. 2) after exposure to RAW 264.7 cells for an increasing length of time. Encapsulated enrofloxacin achieved a concentration of 0.825 μg/mg of protein within 15 min and continued to accumulate intracellularly to a maximum concentration of 0.886 μg/mg of protein after 0.5 h, while the free enrofloxacin reached a maximum intracellular concentration of 0.031 μg/mg of protein at 15 min.

### Effect of size on the cellular uptake of encapsulated enrofloxacin

**Figure 3:**
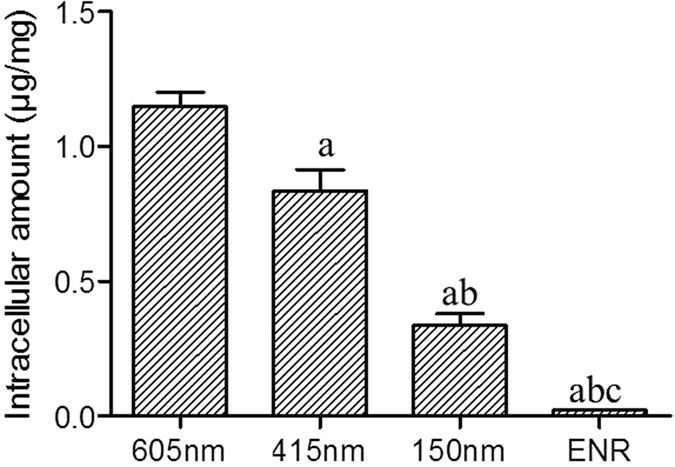
Effect of size on the uptake of docosanoic acid SLNs entrapped enrofloxacin in RAW.264.7 cells. ^a^Statistical significances compared with 605 nm are p < 0.05. ^b^Statistical significances compared with 415 nm are p < 0.05. ^C^Statistical significances compared with 150 nm are p < 0.05. The 605 nm SLNs were prepared with 0.2 g enrofloxacin, 1.8 g docosanoic acid and 10 ml 1% PVA by using 3 mm sonication probes with 60% amplitude. The 415 nm SLNs were prepared with 0.2 g enrofloxacin, 1.8 g docosanoic acid, and 20 ml 2% PVA by using 6 mm sonication probes with 60% amplitude. The 150 nm SLNs were prepared with 0.2 g enrofloxacin, 1.8 g docosanoic acid, and 30 ml 4% PVA by using 30 mm sonication probes with 80% amplitude.

The enrofloxacin in RAW 264.7 cells was enhanced by an increase in the particle sizes of docosanoic acid SLNs, when incubated for 0.5 h. Docosanoic acid nanoparticles of 605, 415, and 150 nm resulted in intracellular enrofloxacin concentrations of 1.147, 0.834 and 0.336 μg/mg of protein, respectively (Fig. 3).

### Effect of zeta potential on the cellular uptake of enrofloxacin

**Figure 4:**
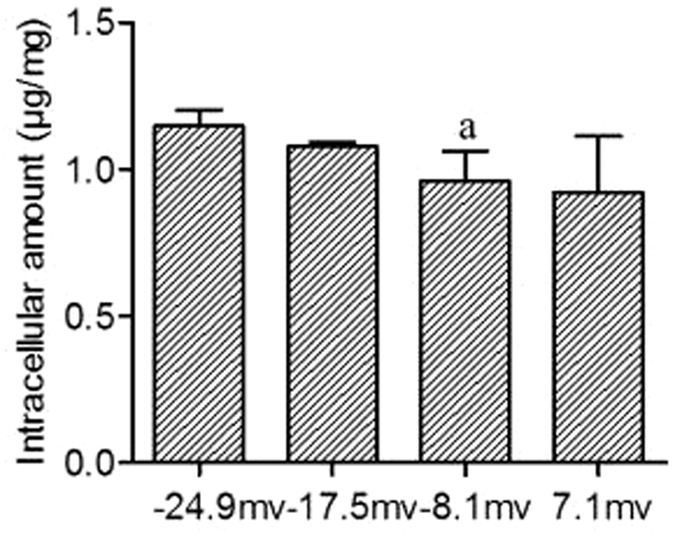
Effect of zeta potential on the uptake of docosanoic acid SLNs with similar sizes entrapped enrofloxacin in RAW.264.7 cells. The −24.9 mv SLNs were prepared with 0.2 g enrofloxacin, 1.8 g docosanoic acid, and 10 ml 1% PVA. The −17.5 mv SLNs were prepared with 0.2 g enrofloxacin, 1.8 g docosanoic acid, and 20 ml 2% PVA and 0.5% DDAC. The −8.1 mv SLNs were prepared with 0.2 g enrofloxacin, 1.8 g docosanoic acid, and 20 ml 2% PVA and 2% DDAC. The 7.1 mv SLNs with 501 nm were prepared with 0.2 g enrofloxacin, 1.8 g docosanoic acid, and 20 ml 2% PVA and 3% DDAC. ^a^Statistical significances compared with −24.9 mv are p < 0.05.

**Figure 5:**
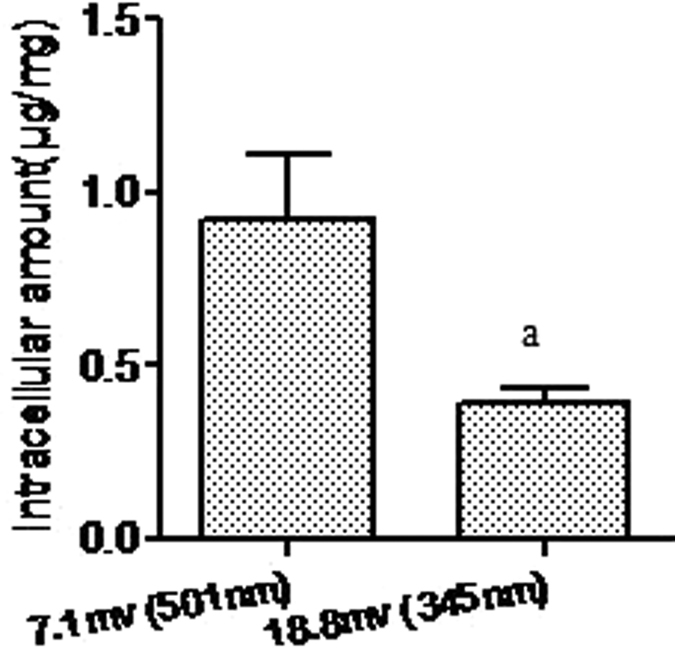
The uptake of different zeta potential and size docosanoic acid SLNs entrapped enrofloxacin in RAW.264.7 cells. The SLNs with 7.1 mv and 501 nm were prepared with 0.2 g enrofloxacin, 1.8 g docosanoic acid, and 20 ml 2% PVA and 3% DDAC. The SLNs with 18.8 mv and 345 nm were prepared with 0.2 g enrofloxacin, 1.8 g docosanoic acid, and 20 ml 2% PVA and 4% DDAC. ^a^Statistical significances compared with 7.1 mv are p < 0.05.

The zeta potential of docosanoic acid SLNs influenced the uptake of entrapped enrofloxacin in RAW 264.7 cells after an experimental period of 0.5 h (Fig. 4). The intracellular enrofloxacin decreased from 1.147 to 0.960 μg/mg of protein due to a decrease in the absolute value of the nanoparticle surface charge from 24.9 to 8.1 mv when the cells were incubated with nanoparticles of equivalent size. The intracellular delivery efficacy of 7.1 mv nanoparticles with an average size of 501.3 nm was the same as that of −8.1 mv SLNs of 532.1 nm in size (Fig. 4). These results demonstrated that the net charge, rather than whether the charge was positive or negative, influenced the intracellular delivery of docosanoic acid SLN-encapsulated enrofloxacin. In addition, the intracellular content of 7.1 mv docosanoic acid nanoparticles of 501.3 nm in size was 2.35 times higher than that of 18.8 mv SLNs of 345.2 nm in size (Fig. 5), which suggested that particle size played a more important role than zeta potential in cellular uptake.

### Intracellular elimination of encapsulated enrofloxacin

**Figure 6:**
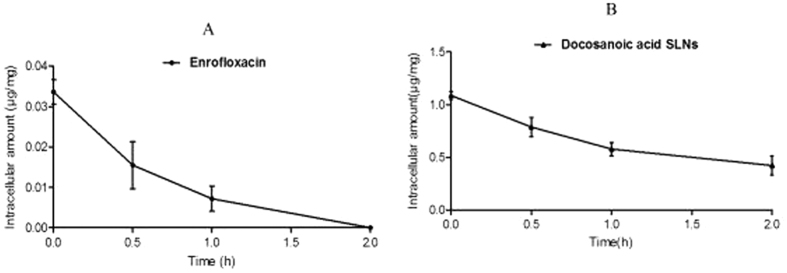
The intracellular elimination kinetics of enrofloxacin. (**A**) Free enrofloxacin; (**B**) Docosanoic acid encapsulated enrofloxacin. The enrofloxacin-loaded SLNs with 605 nm and −24.9 mv were prepared with 0.2 g enrofloxacin, 1.8 g fatty acid and 20 ml 2% PVA.

The intracellular elimination of optimum (605 nm and −24.9 mv) docosanoic acid SLN-encapsulated enrofloxacin was determined by removing the extracellular drug after exposure to the RAW 264.7 cells for 0.5 h. The intracellular content of free enrofloxacin decreased by 53.87% and 78.57% after 0.5 and 1 h, respectively, while the docosanoic acid SLN-encapsulated enrofloxacin was only reduced by 27.53% and 46.72% (Fig. 6). After incubation for 2 h, the intracellular encapsulated drug remained at a concentration of 0.423 μg/mg, while the free drugs were no longer detectable in the cells. These results showed that the docosanoic acid SLNs’ payload of enrofloxacin was eliminated from the cells much more slowly than the free drug.

### The activity of SLN-encapsulated enrofloxacin against intracellular Salmonella

**Figure 7:**
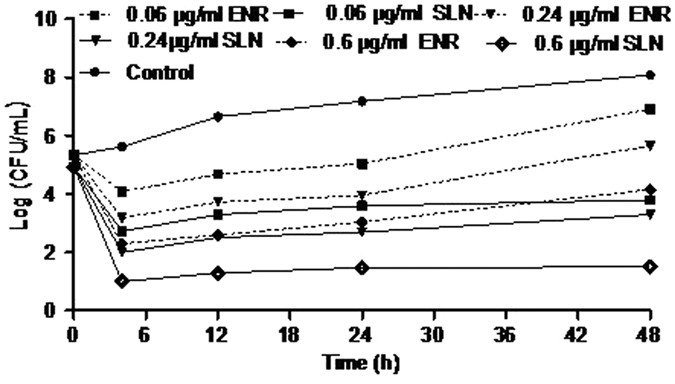
Inhibition curve of enrofloxacin and docosanoic acid SLN entrapped enrofloxacin against intracellular *salmonella CVCC541*. ENR: Free enrofloxacin; DAS: Docosanoic acid SLNs. The enrofloxacin-loaded SLNswith 605 nm and −24.9 mv were prepared with 0.2 g enrofloxacin, 1.8 g fatty acid and 10 ml 1% PVA.

Enrofloxacin-loaded docosanoic acid SLNs of 605 nm and −24.9 mv were more effective against intracellular *Salmonella CVCC541* than free enrofloxacin at three different concentrations: 0.06, 0.24 and 0.6 μg/mL. The 0.24 μg/mL encapsulated enrofloxacin exhibited stronger antibacterial activity than 0.6 μg/mL free enrofloxacin throughout the incubation period. As the incubation time increased, the inhibitory effect of docosanoic acid SLN-encapsulated enrofloxacin was more significant than free enrofloxacin (Fig. 7). At 48 h, the intracellular colony logarithmic value of the 0.6 μg/mL free enrofloxacin group (4.15 colony forming units (CFU)/mL) was greater than that of the 0.06 μg/mL encapsulated enrofloxacin group (3.80 CFU/mL). The 0.6 μg/mL encapsulated enrofloxacin could decrease the intracellular bacteria by 99.97%, thus reaching the minimum bactericidal concentration.

## Discussion

The strong antibacterial potency and efficient permeation of enrofloxacin into cells makes it an appealing choice for the treatment of infections caused by *Salmonella*, but it always fails to completely eradicate intracellular *Salmonella* infection in animals due to its rapid efflux from cells when the extracellular concentration falls^6^,^16^,^17^. In order to enhance the intracellular delivery of this drug, enrofloxacin-loaded docosanoic acid SLNs were prepared and examined in this study. The docosanoic acid SLN-encapsulated enrofloxacin rapidly reached a much higher maximum intracellular concentration than the free enrofloxacin. The docosanoic acid SLNs were 27.06–37.71 times more efficient in the delivery of their enrofloxacin payload into RAW 264.7 cells than the free drug, although the rapid equilibration of the free drug within the cells suggests that the plasma membrane does not present a huge barrier to enrofloxacin penetration. The significant increase in intracellular accumulation will increase the efficacy of encapsulated enrofloxacin relative to the free drug.

A growing body of research demonstrates that the properties of nanoparticles can affect their uptake and that of their encapsulated antimicrobial agents^14^. Docosanoic acid SLNs of different sizes and zeta potentials were prepared by the adjustment of ultrasonic power, ultrasonic time, and the type and concentration of surfactant used. These variations were used to find the common and decisive factors of docosanoic acid nanoparticles in mediating their cellular uptake and thus to maximize intracellular accumulation of encapsulated enrofloxacin. The size of nanoparticles was found to significantly affect the cellular uptake of docosanoic acid SLN-encapsulated enrofloxacin. The intracellular concentrations were significantly enhanced when size was increased from 150 to 605 nm, which might be due to the fact that cellular uptake of nanoparticles in phagocytes predominantly depends on phagocytosis when their size increases beyond 100 nm^14^. In most cases, the phagocytosis rate of polymeric nanoparticles and liposomes is enhanced when particle size increases over the range of 100–1000 nm and becomes constant at over 1000 nm^13^,^14^,^18^,^19^. Therefore, the PDI of docosanoic acid SLNs, which reflects the uniformity of nanoparticle sizes, should be as low as possible in the preparation process.

The surface charge of docosanoic acid nanoparticles was also found to be an important factor in determining the uptake behavior of their encapsulated drugs, although whether the charge was positive or negative did not have an effect. For the three different anionic docosanoic acid SLNs, the cellular uptake of enrofloxacin was positively correlated with the net charge of the nanoparticles. This is consistent with other reports that the cellular accumulation of azithromycin and ciprofloxacin increased in proportion to the liposomal negative charge^20^,^21^. The preferential cellular uptake of highly negatively charged nanoparticles is probably due to the high charge density areas at the cell surface, which can mediate the non-specific interactions with non-specific receptors by electrostatic interactions^22^, especially with the type B scavenger receptor^23^. The higher intracellular content of docosanoic acid nanoparticles of 7.1 mv and 501.3 nm than the SLNs of 18.8 mv and 345.2 nm may indicate that size is a more important factor than zeta potential in determining the cellular uptake efficacy of docosanoic acid SLNs. According to the above results, the docosanoic acid SLNs of 605 nm and −24.9 mv were the optimal carriers for intracellular delivery. The intracellular elimination rate of the optimal docosanoic acid nanoparticle-encapsulated enrofloxacin was also significantly slower than the free enrofloxacin.

The enhanced cellular uptake and intracellular retention of encapsulated enrofloxacin suggests that enrofloxacin-loaded docosanoic acid SLNs might be highly effective against intracellular *Salmonella*. Therefore, an intracellular infection with *Salmonella CVCC541* was established to evaluate the selected SLNs. The time-bactericidal curve demonstrated that enrofloxacin-loaded docosanoic acid SLNs displayed 2.5–10 times more effective inhibition against intracellular *Salmonella CVCC541* than free enrofloxacin at the three concentrations: minimal inhibitory concentration (MIC) (0.06 μg/mL), 4MIC (0.24 μg/mL) and 10 MIC (0.6 μg/mL) against *Salmonella CVCC541*. The increase in antibacterial efficacy was not as significant as the increase in the intracellular concentration of the drug. This might be because a substantial portion of the intracellular drug remained associated with the nanoparticles and was sequestered away from the intracellular bacteria^24^. These results are essentially better than other reports in which ciprofloxacin and moxifloxacin bounded to poly (isobutyl cyanoacrylate) and poly (butyl cyanoacrylate) nanoparticles exhibited similar activity to free drugs^24^,^25^.

As the encapsulated enrofloxacin in cells was released from the nanoparticles, the antibacterial effect of enrofloxacin-loaded docosanoic acid SLNs became more pronounced than that of the free drug as the incubation time increased. When the incubation time increased up to 48 h, the intracellular colony (3.80 CFU/mL) of the 0.06 μg/mL encapsulated enrofloxacin group was smaller than that (4.15 CFU/mL) of the 0.6 μg/mL free enrofloxacin group. It is particularly interesting that 0.6 μg/mL docosanoic acid SLN-encapsulated enrofloxacin inhibited 99.97% *Salmonella* growth at 48 h, while the 0.6 μg/mL free enrofloxacin did not inhibit the growth of bacteria during the same experimental period. All of these results indicate that enrofloxacin-loaded docosanoic acid SLNs might be a promising formulation for intracellular *Salmonella* infection therapy. The docosanoic acid SLNs, especially those with a larger size and higher charge, could also be a promising carrier for treating other intracellular bacteria infections.

## Materials and Methods

### Materials

Enrofloxacin of reference standard was purchased from the China Institute of Veterinary Drug Control (Beijing, China). Native enrofloxacin was bought from Wuhan Konglong Century Technology Development Co., Ltd. (Wuhan, China). Docosanoic acid and dimethyldioctadecyl ammonium chloride (DDAC) were purchased from Shanghai Aladdin Biochemical Polytron Technologies Inc. (Shanghai, China). Polyvinyl alcohol (PVA) was obtained from Sigma (St. Louis, MO, USA). Methyl alcohol and acetonitrile with high performance liquid chromatography (HPLC) grade were purchased from Tedia (Ohio, USA). The water for HPLC was prepared with a Milli-Q system (Millipore, Bedford, MA, USA). RAW 264.7 cell lines were obtained from the National Veterinary Drug Residues Reference Laboratory of Huazhong Agricultural University (Wuhan, China). Dulbecco’s modified eagle medium (DMEM, 4.5 g/L of glucose), Dulbecco’s modified eagle medium/Ham’s F-12 mixture (DMEM/F12), penicillin (100 U/mL)-streptomycin (100 mg/mL) and fetal bovine serum (FBS) were purchased from Hyclone Co., Ltd. (Logan City, USA). Radio immunoprecipitation assay (RIPA) cell lysis solution was bought from Shanghai Ruji Biology Technology Co., Ltd. (Shanghai, China). All other reagents and solvents not specified in the text were of analytical grade and commercially available.

### Preparation of enrofloxacin-loaded docosanoic acid SLNs

Enrofloxacin-loaded SLNs were prepared using a hot homogenization and ultrasonication method^15^. The different sizes and zeta potentials of docosanoic acid SLNs were obtained by adjusting the preparation process and the type, concentration and volume of the surfactant. Briefly, 1.8 g docosanoic acid and 0.2 g enrofloxacin were added to a 50 mL tube and put in a boiling water bath. After the drug was dissolved in the melted lipid matrix, different volumes (10, 20 or 30 mL) of 1, 2 or 4% PVA solution, with or without DDAC solution at concentrations of 0.5, 2, 3 or 4%, were preheated in a boiling water bath and poured into the lipid phase under magnetic stirring. The mixture was then sonicated for 8 min using 3, 6 or 30 mm microprobes with 60% or 80% amplitude (VCX 130 Vibra-CellTM, Sonics & Materials, Inc., Newtown, CT, USA) to form a nanoemulsion. The hot nanoemulsion was poured into a certain volume of cold water to obtain a nanoparticle suspension. The nanoparticles were collected by centrifugation at 14,000 rpm (Hitachi Centrifugation CR21G; Hitachi Koki Co., Ltd., Japan) for 60 min at 4 °C, and washed three times with distilled water. The SLNs were suspended in 10 mL distilled water and lyophilized for 48 h (Freeze Dry System; Labconco, USA).

### Atomic force microscopy (AFM)

The morphology of nanoparticles of different sizes was measured using an Aglient 5500 AFM (Agilent Technologies, AZ, USA) under normal atmospheric conditions. In brief, 20 mg samples were suspended in 500 μL distilled water and 2 μL of the suspension was placed on a cover glass. After oven-drying at room temperature, imaging of the samples was performed in contact mode with pyramidal silicon nitride tips.

### Determination of loading capacity and encapsulation efficiency

To determine the enrofloxacin content of the nanoparticles, 10 mg freeze-dried SLNs was added to a 15 mL tube containing 10 mL acetonitrile/water solution (v/v; 1:1) and put in a boiling water bath for 20 min to destroy the nanoparticles so that the drug was completely released. The nanoparticle suspension after heating was added to the volume of 10 mL and centrifuged at 8,000 rpm (Hitachi Centrifugation CR21G; Hitachi Koki Co., Ltd., Japan) for 10 min. The supernatant was diluted 100-fold and injected into Waters 2695 series HPLC equipped with a UV detector (Waters Corp., Milford, MA, USA) for analysis after filtration. The assay was repeated three times using different samples from independent preparations. The loading capacity (LC) and encapsulation efficiency (EE) were defined as follows:


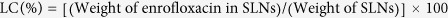



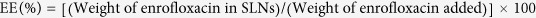


### Determination of size, polydispersity index (PDI) and zeta potential

The size, PDI and zeta potential of different enrofloxacin-loaded SLNs were measured by photon correlation spectroscopy (PCS) by using Zetasizer ZX3600 (Malvern Instruments, UK) at 25 °C. The samples were suspended in distilled water by ultrasonication for 5 s at 0 °C to remove the air bubbles and break up the agglomerates. The concentration of the sample was 2.7 mg/mL for the tests of size and PDI, and 0.3 mg/mL for the test of zeta potential, in order to get the optimum kilo counts per second of 20–400 for the measurements^14^. All measurements were repeated in triplicate by using different samples from independent preparations.

### Cell culture

The RAW 264.7 cells were grown in culture flasks (Corning Costar Co., Ltd., NY, USA) containing DMEM supplemented with 10% (v/v) FBS, 1% (v/v) L-Glutamine solution and 1% (v/v) penicillin-streptomycin, at 37 °C in an ambient atmosphere with 5% CO_2_. For routine maintenance, the cell medium was changed every 24 h and the cells were sub-cultured with 0.25% trypsin −0.02% ethylene diamine tetraacetic acid (EDTA) solution after reaching 80–90% confluence.

### Determination of cellular uptake of encapsulated enrofloxacin

For the cellular uptake experiment, the cells were seeded at 1 × 10^5^ cells/cm^2^ onto 6-well culture plates in volumes of 2 mL. When the cells reached about 80–90% confluence, the medium was replaced with pH 7.4 Hanks’ balanced salt solution (HBSS) and the cells were pre-incubated at 37 °C for 1 h. After pre-incubation, the cells were incubated with 2 mL fresh incubation medium containing 10 μg/mL free enrofloxacin or different sizes and zeta potentials of docosanoic acid SLN-encapsulated enrofloxacin for 0.083, 0.25, 0.5 and 1 h. The surface of RAW 264.7 cells was quickly rinsed three times with Phosphate buffer (PBS) at 4 °C to remove the extracellular drug. The washed cells were lysed using 150 μL RIPA cell lysis solutions and collected with 1 mL deionized water for each well. The collected cells were sonicated with an ultrasonic cell disruption system (VCX130; Sonics & Materials, Inc., USA) for 30 s. Subsequently, 5 μL cell lysate was used to detect the protein content with the bicinchoninic acid (BCA) method. The remainder of the cell lysate was deproteinized using 1 mL methanol under vortex mixing for 2 min and centrifugation at 12,000 rpm for 15 min at 4 °C. The supernatant was evaporated to dryness under a nitrogen evaporator (N-EVAP112; Organomation Associates Inc., USA) at 30 °C. The concentrates were dissolved with 500 μL mobile phase and injected into HPLC vials for analysis with a fixed injection volume of 40 μL.

### Determination of intracellular elimination of encapsulated enrofloxacin

To determine the intracellular drug elimination process, the confluent RAW 264.7 cells in each well of the 6-well culture plates were exposed to 10 μg/mL free enrofloxacin and docosanoic acid SLN-encapsulated enrofloxacin (in SLNs of 605 nm and −24.9 mv) for 0.5 h. Afterwards, the medium (without FBS, L-Glutamine solution and penicillin-streptomycin) containing enrofloxacin was replaced by the blank basic medium and the cells continued to incubate. After continued incubation for 0.5, 1, and 2 h, the cell surface was quickly rinsed with 4 °C PBS three times to remove the extracellular drug, and the intracellular enrofloxacin was measured by HPLC.

### Determination of enrofloxacin and protein levels

Enrofloxacin was determined using a Waters 2695 series HPLC and a Waters 2587 UV detector set at a wavelength of 278 nm. The chromatographic separation was achieved with an analytical ZORBAX SB-2 C_18_ column (250 × 4.6 mm, i.d. 5 μm; Agilent Technology, USA) at 30 °C. The mobile phase was acetonitrile and 0.1% formic acid solution with the proportion of 14/86 (v/v) and a flow rate of 1.0 mL/min. The working curve for the uptake experiment was y = 189.72 × −902.22 over the concentration range of 40–1000 μg/L (r^2^ = 0.9994). The recovery of the three different concentrations (40, 500 and 1000 μg/L) was 88.2%, 93.6% and 97.5% respectively, from RAW 264.7 lysate. The intra-day and inter-day relative standard deviations (RSD) were lower than 7%. The limit of detection (LOD) and quantification (LOQ) were 20 and 40 μg/L, respectively.

The protein concentration was measured using a bicinchoninic acid (BCA) protein assay kit with bovine serum albumin (BSA) as the standard. In brief, after centrifugation, 5 μL cell lysate was added to 200 μL BCA reagents in 96-well plates and the absorbance was read at 562 nm with a Multiskan spectrum microplate reader (Elx800; Bio-tek instrument, Inc., USA). The BSA linear equation was y = 0.8984 × +0.1229 over the linear range of 0.025–0.5 mg/mL (r^2^ = 0.9994).

### Determination of the activity of encapsulated enrofloxacin against intracellular

#### Salmonella

To determine the antimicrobial effect of enrofloxacin against intracellular bacteria, RAW 264.7 cells in 24-well culture plates (10^6^ cells per well) were infected with 10^7^ CFU/mL of *Salmonella CVCC541* for 1 h. Afterwards, the medium containing bacteria was removed and the cells were incubated with 0.5 mL of 100 μg/mL gentamicin for 0.5 h to completely kill the extracellular bacteria. Extracellular gentamicin was removed with 4 °C PBS three times and the cells were incubated with DMEM basic medium for 4 h to establish the infection model. Nanoparticles of 605 nm and −24.9 mv with encapsulated or free enrofloxacin of 0.06, 0.24 and 0.6 μg/mL were added to the cultures and remained there for 0, 4, 12, 24 and 48 h. At fixed time points, the extracellular bacteria were removed by three consecutive washes with PBS at 4 °C, and the intracellular viable *Salmonella* counts (CFU/mL) were performed by plating serial dilutions of the cell lysates and counting the number of colonies after incubation at 37 °C for 24 h. The time-kill curve was created by plotting average counts as a function of time.

#### Statistical Analysis

Data were expressed as mean ± S.D. and assessed using one-way analysis of variance (ANOVA) by GraphPad Prism. Significance was evaluated at *p*-value of 0.05, respectively.

## Additional Information

**How to cite this article**: Xie, S. *et al*. Enhanced intracellular delivery and antibacterial efficacy of enrofloxacin-loaded docosanoic acid solid lipid nanoparticles against intracellular *Salmonella. Sci. Rep.* **7**, 41104; doi: 10.1038/srep41104 (2017).

**Publisher's note:** Springer Nature remains neutral with regard to jurisdictional claims in published maps and institutional affiliations.

## Figures and Tables

**Table 1:** Physicochemical characteristics of enrofloxacin-loaded docosanoic acid SLNs with different polyvinyl alcohol concentration and preparation process (mean ± S.D., n = 3).

Surfactant		Probe Sizes (mm)	MD (nm)	PDI	ZP (mv)	EE (%)	LC (%)
PVA concentration	PVA volume (mL)						
1%	10	3	605.0 ± 4.9	0.241 ± 0.076	−24.9 ± 0.7	95.9 ± 1.6	9.3 ± 0.2
2%	20	6	414.5 ± 3.8^a^	0.265 ± 0.019	−22.1 ± 0.1^a^	86.6 ± 1.7^a^	8.6 ± 0.2
4%	30	30	150.1 ± 1.9^a^,^b^	0.184 ± 0.018^b^	−13.1 ± 1.0^a^,^b^	59.2 ± 3.5^a^,^b^	5.8 ± 0.4^a^,^b^

EE: Encapsulation efficiency; LC: Loading capacity; MD: Mean diameter; PDI: Polydispersity index; ZP: Zeta potential.

^a^Statistical significances compared with 1% PVA are p < 0.05.

^b^Statistical significances compared with 2% PVA are p < 0.05.

**Table 2:** Physicochemical characteristics of enrofloxacin-loaded docosanoic acid SLNs with different concentration of dimethyldioctadecyl ammonium chloride (mean ± S.D., n = 3).

Surfactant		Probe Sizes (mm)	MD (nm)	PDI	ZP (mv)	EE (%)	LC(%)
Concentration	Volume (mL)						
2%PVA	20	6	414.5 ± 3.8	0.265 ± 0.019	−22.1 ± 0.1	86.6 ± 1.7	8.6 ± 0.2
2%PVA + 0.5%DDAC	20	6	617.5 ± 7.1^a^	0.458 ± 0.010^a^	−17.5 ± 0.6^a^	42.8 ± 2.3^a^	4.4 ± 0.2^a^
2%PVA + 2%DDAC	20	6	532.1 ± 10.0^a^,^b^	0.461 ± 0.058^a^	−8.1 ± 0.4^a^,^b^	41.2 ± 0.8^a^	4.3 ± 0.1^a^
2%PVA + 3%DDAC	20	6	501.3 ± 16.6^a^,^b^	0.417 ± 0.016^a^,^b^	7.1 ± 0.5^a^,^b^,^c^	46.7 ± 2.4^a^	4.8 ± 0.3^a^
2%PVA + 4%DDAC	20	6	345.2 ± 9.6^a^,^b^,^c^,^d^	0.393 ± 0.011^a^,^b^	18.8 ± 0.2^a^,^b^,^c^,^d^	45.6 ± 1.8^a^	4.7 ± 0.2^a^

DDAC: Dimethyldioctadecyl ammonium chloride; EE: Encapsulation efficiency; LC: Loading capacity; MD: Mean diameter; PDI: Polydispersity index; ZP: Zeta potential.

^a^Statistical significances compared with 2% PVA are p < 0.05.

^b^Statistical significances compared with 2% PVA + 0.5% DDAC are p < 0.05.

^c^Statistical significances compared with 2% PVA + 2% DDAC are p < 0.05.

^d^Statistical significances compared with 2% PVA + 3% DDAC are p < 0.05.
